# Valorization of fecal sludge stabilization via vermicomposting in microcosm enriched substrates using organic soils for vermicompost production

**DOI:** 10.1016/j.heliyon.2021.e06422

**Published:** 2021-03-08

**Authors:** Rapheal Nsiah-Gyambibi, Helen Michelle Korkor Essandoh, Nana Yaw Asiedu, Bernard Fei-Baffoe

**Affiliations:** a Regional Water and Environmental Sanitation Centre, Kumasi. Department of Civil Engineering, College of Engineering Kwame Nkrumah University of Science and Technology, UPO, Kumasi, Ghana; b Department of Chemical Engineering, College of Engineering, Kwame Nkrumah University of Science and Technology, PMB, UPO, Kumasi, Ghana; c Department of Theoretical and Applied Biology, College of Science, Kwame Nkrumah University of Science and Technology, PMB, UPO, Kumasi, Ghana

**Keywords:** Fecal sludge, Substrate, *Eisenia fetida*, Vermicompost, Enzymes

## Abstract

High generation of fecal sludge without proper treatment is a major sanitation problem. A key step in curbing this problem is producing value-added resources such as vermicompost from fecal sludge through substrate enrichment. Substrate enrichment is a vermicomposting technique that involves augmenting vermibed substrates with organic rich materials to provide additional nutrients, as well as underlying layers needed for microcosm development to produce desirable vermicompost. The aim of this study was to investigate effects of substrate enrichment with organic soils (black soil, red laterite soil and sandy soil) combined with coconut coir as bulking material, on the fecal sludge vermicomposting process and quality of the end-product. The purpose of the study was to promote the development of highly nutritive vermicompost from fecal sludge using substrate enrichment as a low-cost innovative vermicomposting technique. The enriched substrates were prepared with 160g of coconut coir, 120g of fecal matter (65–70% dry matter) and 80g of organic soil. The treatments were labelled T_1_, T_2_ and T_3_ representing systems containing black soil, red laterite soil and sandy soil respectively. The control treatment (T_4_) contained no soil. Triplicate treatments were setup and about 20 3-week old clitellated earthworms of the species *Eisenia. fetida* with live weights ranging from 255 to 275mg, released into each system for vermicomposting over a period of 12 weeks. Physicochemical parameters such as pH, Organic Carbon (C_org_), Total Nitrogen (N_tot_), Available Phosphorus (P_avail_), Exchangeable Calcium (Ca_exch_), Iron (Fe), Lead (Pb) and Aluminium (Al) were determined for both the fecal sludge and the vermicompost. The vermicompost in the setup with black soil (T_1_) showed the highest C_org_ mineralization and N_tot_, P_avail_ and Ca_exch_ enhancement followed by T_2_, T_3_ and T_4_. Treatment T_1_ also resulted in the lowest concentration of Fe, Pb and Al in the vermicompost. Concentrations of these heavy metals were found to be higher in the other treatments in increasing order of T_2_, T_3_ and T_4_. Less than 16% earthworm mortality was recorded in all treatments except T_4_, in which the mortality was about 38% (38.33 ± 13.74). The enriched substrates were therefore found to provide a more suitable microclimate for earthworm development and produced vermicompost with high nutrient content. However, a more comprehensive study on metal accumulation in the earthworm tissues as a potential metal contaminant is needed to establish a strong hypothesis in the safe use of earthworms for this vermicomposting technique.

## 1 Introduction

The volume of fecal sludge produced yearly is increasing due to population growth and urbanization. This presents a major sanitation problem for developing countries due to inadequate and inappropriate fecal sludge treatment techniques employed (Onodera et al., 2014). A large percentage of the fecal sludge is poorly disposed of with little or no treatment. Non-treatment and improper disposal of fecal sludge accounts for roughly 40% of the total amount of greenhouse gases (GHG) discharged, with the attendant negative impacts on the environment and climate change. Improper disposal of fecal sludge also has detrimental effects on human health due to the presence of contaminants, such as organic compounds, pathogens and heavy metals (Fernandes et al., 2005). With proper treatment, these contaminants are removed or reduced to the safest thresholds prior to disposal or reuse.

Nevertheless, fecal sludge contains beneficial nutrients that can be enhanced and harnessed into valuable resources for agricultural utilization such as soil conditioners and fertilizers (Bettiol, 2004). Practices that will ensure the treatment of fecal sludge for safe disposal or reuse therefore have the potential to be highly embraced (Capodaglio et al., 2017). Studies have identified vermicomposting as an economical bioconversion and a fast-evolving fecal sludge utilization method to produce value-added resources (Rupani et al., 2013). It is a hygienic approach capable of generating value-added vermicompost with low phytotoxicity, high nutrient enhancement, greater fertilizer value and additional production of earthworms at a lower processing time (Lorimor et al., 2001). Vermicompost is also rich in plant nutrients, microbial population, soil enzymes and humic acids that are needed for improving the fertility of soil and increasing the yield of agricultural produce (Aira et al., 2002). There is considerable evidence that contaminants are removed in vermicomposting (Edwards and Bohlen, 1996). Ansa et al. (2017) also report that fecal sludge stabilization via vermicomposting effectively reduces pathogen concentrations to very low levels to attain hygienically safe composts for reuse. Vermicomposting stabilizes fecal sludge into vermicompost through biological aerobic decomposition by earthworms, bacteria, fungi and actinomycetes (Erdal and Ekinci, 2017 ). While microflora and fauna condition the waste for composting, the earthworms maintain aerobic conditions in the waste through their burrowing activities. The earthworms eat, grind, and digest the organic wastes converting it into vermicompost. The vermicompost produced is mostly much finer, more stable, homogenous and humified, and is a microbially active material with desirable aesthetics and reduced levels of contaminants (Ndegwa and Thompson, 2001). An innovative technique mostly referred to as substrate enrichment is currently being used to produce higher quality and superior vermicompost (Suthar, 2006). The technique involves augmenting vermibed substrates with organic-rich materials to provide additional nutrients and underlying layers needed for the development of the microcosm in the vermicomposting system (Tognetti et al., 2013). Substrate enrichment has been applied in a couple of studies (Benítez et al., 2000; Ndegwa and Thompson, 2001; Suthar, 2007a; Gutiérrez-miceli et al., 2011; Yadav et al., 2011). It has been reported to influence the vermiculture in vermicomposting systems with respect to the growth and reproduction of the earthworms as well as the quality of vermicompost in terms of nutrient enhancement, contaminant removal, microbial biomass and enzyme enhancement (Bassan, 2014; Batham et al., 2013; Rossi, 2009). Brookes (1995) reports that the chemical characteristics of vermicompost largely reflect the quality of the materials present in the vermicomposting substrate.

This study explores the use of organic soils in substrate enrichment to provide an improved technique for vermicomposting of fecal sludge. The rationale for doing so is that reported studies do not definitively account for the impact of organic soils in substrate enrichment for vermicompost production. The unique contribution to knowledge therefore is not only in the use of a cheap and sustainable material (i.e. organic soils) in substrate enrichment, but also to address the knowledge gap in the use of organic soils for enhanced vermicomposting from fecal sludge stabilization. The hypothesis is that the use of organic soils in substrate enrichment for fecal waste vermicomposting would produce an enhanced stabilized vermicompost. This hypothesis is based on the fact that organic soils provide a large pool of soil nutrients (Aira et al., 2002), soil microbial biomass and soil enzymes (Zhang et al., 2000) which are needed for the vermiculture of earthworms and the functioning of the microcosm in the vermicomposting system. The humus and nutrients in organic soils are capable of causing alterations in the composition of the microbial community and the enzymatic activities which play a significant role in contaminant removal (Fernandes et al., 2005; Pontes, 2002). Soil texture and soil organic matter are also suitable characteristics needed in the development and flourishing of microflora and fauna communities that aid in the composting process (Blakemore et al., 1995).

Organic soils with different compositions and properties, it was presumed, would yield end products with varying qualities, hence the approach adopted in this study. Thus, this study aimed to investigate the use of different types of organic soils in substrate enrichment for the production of vermicompost from fecal sludge using an epigeic earthworm species (*Eisenia fetida*). The study investigated the quality of produced vermicompost from application of substrate enrichment treatments. The vermiculture of the earthworms relative to their growth and reproduction were also investigated as these are considered vital parameters that indicate the efficiency of a vermicomposting technique (Gibbs et al., 1996).

## 2 Materials and methods

### 2.1 Collection and treatment of materials

The earthworm species used was *E. fetida* which is an epigeic and a potential waste composting worm (Edwards and Bohlen, 1996). The breeding stock of *E. fetida* was obtained from Green Cycle Technology Company, an earthworm breeder in Ghana. The earthworms were maintained in vermibeds at a temperature of ±25 °C and only mature clitellate worms, of about 3 weeks old, were used for the purposes of this investigation. Fecal sludge, containing 65–70% dry matter, was obtained from an Enviro-loo toilet facility used by a community in the Oforikrom sub-metro of the Kumasi Metropolitan Assembly (KMA) located in Ghana. The toilet facility is located at a distance of about 1 km from the laboratory where the studies were conducted. Enviro-loo toilet facilities are dry sanitation systems that handle human excreta and are affordable, sustainable. The systems are also reputed to have a longer life expectancy and superior cost-effectiveness over their lifetime compared to other systems (Bassan, 2014). Tap water was used to moisten the vermibeds during the vermicomposting process. The organic soils used in the substrate enrichment were categorized as black soil or agricultural soil, red laterite soil and sandy soil based on their characteristics. The black soil was obtained from a vegetable farm and the red laterite and sandy soils from a building construction site. All organic soils were obtained at less than 1m deep. The soils were allowed to open-dry in sunlight for 24 h before use. Coconut coir was used as the bulking material and was obtained locally from a coconut oil mill in the Ejisu Juaben Municipal Assembly (EJMA) of the Ashanti Region of Ghana. The bulking material was also allowed to open-dry in sunlight for 24 h and chopped into small bits (1–20 cm). The characteristics of the fecal sludge, organic soil and bulking material were determined by conducting physico-chemical analysis on samples of these materials. Following Standard Methods of the American Public Health Association (1998), the following physico-chemical analysis were used to characterize the fecal sludge, organic soil and bulking material; eg pH, organic C, total N, available P, exchangeable Ca, Fe, Pb and Al.

### 2.2 Substrate enrichment preparation and treatment design

Vermibeds were constructed in rectangular polyethylene terephthalate plastic containers with dimension of 15cm in length, 15 cm in breadth and 30cm in depth. The base of each vermibed was netted and perforated to allow drainage of excess moisture. The vermibed substrates were prepared with 160g of coconut coir as bulking material which was added to 120g of fresh homogenized fecal matter containing 30–35% moisture. The vermibed substrates were enriched with 80g of organic soil. Three treatments with the organic soils were prepared which were labelled T_1_, T_2_ and T_3_ for setups containing black soil, red laterite soil and sandy soil respectively. A control treatment (T_4_) with no soil in the substrate was also set up. All treatments were setup in triplicate and about 20 3-week old clitellated *E. fetida* (live weight ~255–275 mg) collected from the stock culture were released into each treatment. The moisture level in the treatment substrates was maintained around 45–60% by periodic sprinkling of an adequate quantity of tap water, if required. Vermibeds were placed in a humid and dark room with a temperature of 28.6 ± 0.5 °C and 240g (dry weight basis) fresh sludge was vermicomposted in each treatment for 12 weeks.

### 2.3 Vermiculture

The initial live weight of individual earthworms was determined by weighing them on an electronic scale before stocking them in the vermibeds to determine growth. The earthworms’ growth and cocoon production in each treatment unit were observed in every 14 days for 12 weeks (i.e. at 14, 28, 42, 56, 70 and 84 days. The earthworms and the cocoons were separated from the composted material by hand sorting and washed in tap water to remove adhering material before weighing. The washed earthworms were weighed on a live weight basis in a water filled weighing basin to prevent the worms from desiccating which could have affected their weight. All measured earthworms were returned to their respective containers and the cocoons were counted and introduced into separate bedding. On the basis of obtained data of earthworm biomass and cocoon numbers, other growth parameters of earthworm, i.e. growth rate (mg day^−1^), maximum weight achieved and reproduction rate (cocoonworm^−1^ day^−1^) were calculated.

### 2.4 Quality analysis of vermicompost

To determine the effect of the treatments on the quality of the vermicompost produced, homogenized samples of the vermicompost (25 g dry weight basis) were drawn at day 0 (initial) and at day 84 from each treatment for analysis of the physico-chemical characteristics, heavy metal, microbial biomass, cellulase and urease concentration. The physico-chemical parameters measured included moisture and ash content, pH, organic Carbon (C_org_), total Nitrogen (N_tot_) and available Phosphorus (P_avail_). The moisture and ash content of the vermicompost were determined by drying in an oven at 105 °C for 24 h and 550 °C for 4 h respectively. The pH was measured using digital pH meter (Palintest multi portable meter) in 1/10 (w/v) aqueous solution. C_org_ was determined by standard protocol (APHA, 1998). N_tot_ was measured by Persulfate Digestion Method 10072 of the TNT protocol. C:N ratio was calculated from the measured value of C_org_ and N_tot_ while P_avail_ was determined by standard protocol using Tecator Model Enviroflow- 5012 autoanalyser (Anderson and Ingram, 1993). The exchangeable calcium (Ca_exch_) and heavy metals (Fe, Pb and Al) were analyzed following the atomic absorption spectrophotometer (AAS) method as described by Katz and Jennis (1983). The microbial biomass (nitrogen and carbon) was measured with a method developed by Brookes et al. (1985). Cellulase activity was determined using CM-cellulose method developed by Schinner and von Mersi (1990). Ten (10) grams of biosolid sample was incubated for 24 h at 50 °C in a beaker with 15 ml buffer of 2 M anhydrous sodium acetate and 15 ml of acetate acid. One (1) milliliter of the filtrate was collected into a test tube and further incubated for 15 min at 100 °C with 1 ml reagent A (16g Na_2_CO_3_ + 0.9 g KCN dilute to 1000 ml) and 1 ml reagent B (0.5 g K_3_Fe(CN)_6_ dilute to 1000 ml). The incubated filtrate was mixed with 5 ml of reagent C (1.5g NH_4_Fe(SO_4_)_2_ + 1 g CH_3_(CH_2_)_11_OSO_3_Na) and allowed to stand for an hour prior spectrophotometer reading at 690 nm. Results were expressed as μg Glucose equivalents∙g^−1^ dry matter∙24 h^-1^. Urease activity was determined with a method developed by Tabatabai and Bremner (1972). Five (5) grams of biosolid sample was incubated in a beaker with 2.5 ml of 0.72 M urea substrate solution and 20 ml of 0.1 M borate buffer for 2 h at 37 °C. Thirty (30) millilitres of 2 M KCl was added to the incubated sample and allowed to spin on a rotary shaker for 30 min. The sample was then filtered and 1 ml of filtrate was mixed with 5 ml of reagent A (1.2 g NaOH +17 g HOC_6_H_4_COONa +0.12 g Na_2_[Fe(CN)_5_NO] diluted to 300 ml with distilled water. Suspension was allowed to stand for 30 min before taking to the spectrophotometer to be read at 690 nm. Results were expressed as μg N–NH_4_∙g^−1^ dry matter hours^−1^.

Chemical compositions and metal contents of substrates at startup used for experimentation (mean ± S.D.; n = 3) are presented in Table 2.

Microbial biomass and enzyme contents of substrates used for experimentation (mean ± S.D.; n = 3) at startup are presented in Table 3.

### 2.5 Statistical analysis

The experimental design was a complete randomized block design (CRBD) where treatments were randomly assigned in triplicates. One-way ANOVA using the Student-Newman-Keuls (S–N–K) post hoc pairwise multiple comparison procedure was used to analyze the significant difference in the physio-chemical parameters between treatments. A Spearman and Pearson correlation matrix was used to determine the correlation coefficients between variables. Dunn's multiple-ranged test was also performed to identify the homogeneous type of the bedding material with respect to earthworm's growth parameters (earthworm weight gain, individual growth rate, total number of cocoons, cocoon production rate, and total population mortality, etc.). A Mann-Whitney paired-sample t-test was performed between control (compost without worms) and experiment (vermicompost with earthworms) for different chemical parameters.

## 3 Results and discussion

### 3.1 Physio-chemical changes during vermicomposting

The organic soils and the coconut coir were acidic with high organic carbon (>400 g kg^−1^) and this could be attributed to the sources of deposits that may contain acidic and carbon elements. The high organic carbon in these materials could explain the high C/N ratios recorded as shown in Table 1. The Ca_exch_ as well as Fe, Pb and Al were also high and this could again be due to the sources of deposits. The fecal sludge however was more alkaline with a pH range of 7.2–7.5 which is attributable to bicarbonates that may be present in the waste (Bassan, 2014). The fecal sludge contained some levels of Ca_exch_, Fe, Pb and Al but concentrations were lower compared to the organic soils. Treatment substrates at startups showed no significant differences in the pH, N_tot_, P_avail,_ and C:N but there were significant differences in the C_org,_ Ca_exch,_ Fe, Pb and Al as presented in Table 2. Similarly, treatment substrates at startups showed no significant differences in the microbial N-biomass and Cellulase but recorded significant differences in the microbial C-biomass and Urease as presented in Table 3.

**Table 1:** Characteristics of fecal sludge, organic soils and bulking material (mean ± S.D.; n = 3).

Parameter	Black soil	Red laterite soil	Sandy soil	Fecal sludge	Coconut coir
pH	6.82 ± 0.02	6.50 ± 0.03	6.70 ± 0.02	7.48 ± 0.08	6.71 ± 0.02
C_ogr_ (g kg^−1^)	496.20 ± 0.80	466.20 ± 0.51	445.10 ± 0.20	256.06 ± 0.7	421.13 ± 1.10
N_tot_ (g kg^−1^)	20.92 ± 1.28	20.87 ± 0.55	21.74 ± 1.65	25.75 ± 1.18	23.97 ± 1.59
P_avail_ (g kg^−1^)	27.16 ± 0.50	18.10 ± 0.10	16.30 ± 0.45	35.90 ± 0.29	16.48 ± 0.15
C:N ratio	14.15 ± 0.18	23.33 ± 0.21	21.17 ± 0.20	10.78 ± 0.14	18.53 ± 0.15
Ca_exch_ (g kg^−1^)	59.40 ± 1.22	40.31 ± 1.22	32.40 ± 1.25	37.44 ± .80	43.36 ± 1.20
Fe (mg kg^−1^)	541.18 ± 0.80	651.35 ± 1.20	440.55 ± 0.90	322.50 ± 0.50	139.23 ± 0.73
Pb (mg kg^−1^)	29.20 ± 0.20	30.60 ± 1.20	19.51 ± 1.25	21.9 ± 0.40	11.9 ± 0.14
Al (mg kg^−1^)	275.80 ± 0.22	380.42 ± 0.60	123.72 ± 1.00	84.10 ± 0.30	51.10 ± 0.12
Clay<0.002 mm (%)	1.12 ± 0.22	23.13	-		
Silt 0.002–0.06 mm (%)	3.59 ± 0.20	27.69 ± 0.10	-		
Sand 0.06–2 mm (%)	88.68 ± 0.10	14.74 ± 0.30	99.51 ± 0.22		
Gravel >2 mm (%)	3.38 ± 0.10	29.40 ± 0.10	0.49 ± 0.10		
Liquid Limit (%)	46.8 ± 0.20	59.4 ± 0.20	32.0 ± 0.10		
Plastic Limit (%)	19.3 ± 0.30	26.4 ± 0.20	14.3 ± 0.20		
USCS Classification	SP-SC	CH	SW		
Classification meaning	Poorly graded sand with clay	Highly plasticity	Highly graded sand

**Table 2:** Chemical composition of substrates at startup (mean ± S.D.; n = 3).

Treatment	pH	C_org_ (g kg^−1^)	N_tot_ (g kg^−1^)	P_avail_ (g kg^−1^)	C:N ratio	Ca_exch_ (g kg^−1^)	Fe (mg kg^−1^)	Pb (mg kg^−1^)	Al (mg kg^−1^)
T_1_	7.52 ± 0.04a	334.69 ± 0.56d	23.26 ± 0.64a	25.51 ± 0.25a	13.97 ± 0.39a	47.37 ± 0.05a	254.40 ± 0.62a	19.22 ± 0.50d	60.20 ± 0.30a
T_2_	7.52 ± 0.03a	323.55 ± 0.96c	24.23 ± 0.68a	25.97 ± 0.15a	13.36 ± 0.33a	54.21 ± 0.02d	369.50 ± 0.60d	40.41 ± 0.20c	81.60 ± 0.20d
T_3_	7.51 ± 0.02a	314.65 ± 0.53b	23.33 ± 1.15a	25.74 ± 0.40a	13.94 ± 0.70a	47.75 ± 0.08c	262.20 ± 0.22c	12.20 ± 0.20b	65.46 ± 0.34c
T_4_	7.50 ± 0.06a	305.78 ± 0.64a	23.41 ± 0.67a	25.47 ± 0.40a	13.07 ± 0.40a	45.80 ± 0.05b	68.60 ± 0.40b	8.50 ± 0.20a	19.20 ± 0.50b

Mean values followed by different letters are statistically different (ANOVA, Dunns multiple-ranged test; P < 0.05.

**Table 3:** Microbial biomass and enzyme concentration of substrates used for experimentation (mean ± S.D.; n = 3) at startup.

Treatment^a^	Carbon (μg C g^−1^ dry mass)	Nitrogen (μg N g^−1^ dry mass)	Cellulase (μg Glucose equivalents∙g^−1^dry matter∙24 h^-1^)	Urease (μg N–NH_4_ g^−1^ dry matter hr^−1^)
T_1_	360.81 ± 6.60b	114.60 ± 0.50a	21.20 ± 0.60a	2.90 ± 1.55b
T_2_	280.40 ± 3.50ab	56.70 ± 0.62a	18.32 ± 1.40a	2.80 ± 1.20b
T_3_	253.50 ± 3.06a	50.40 ± 0.55a	16.80 ± 1.30a	2.50 ± 1.40b
T_4_	261.99 ± 5.91a	47.77 ± 0.96a	15.07 ± 4.29a	2.80 ± 0.82a

Mean values followed by different letters are statistically different (ANOVA, Dunns multiple-ranged test; P < 0.05.

The results show a pH reduction within the range of 5.7–9.0% in the vermicompost produced from the treatments (Table 4). The pH reduction is similar to observations made in previous studies (Suthar, 2009; Ndegwa and Thompson, 2001). pH reduction in vermicomposting is mostly related to the mineralization and bioconversion of the organic material into CO_2_ and intermediate species of organic acids by the microbial decomposition (Ndegwa and Thompson, 2001). There were significant differences in C_org_ (F = 490.6, P < 0.001), N_tot_ (F = 24.30, P < 0.001), P_avail_ (F = 450.5, P < 0.001), and Ca_exch_ (F = 1201, P < 0.001) between the produced vermicompost the initial substrates. Organic Carbon (C_org_) content was observed to reduce in the vermicompost from all treatments compared to the initial substrates (Table 4). This showed that vermicomposting of organic substrates using *E. fetida* mediated C_org_ mineralization. This observed C_org_ mineralization is consistent with previous reports (Thomas et al., 2013; Suthar, 2009; Kala et al., 2009; Suthar and Singh, 2008a). C_org_ loss or mineralization in vermicomposting demonstrates stabilization of the organic waste, likely caused by the activities of earthworms where they fragment and homogenize the ingested organic material through muscular action of their foregut, adding mucus and enzymes to the ingested material, digesting and assimilating the carbon content and finally egesting the reduced C_org_ material as vermicastes to produce the vermicompost (Chang and Chen, 2010; Domínguez, 2004). The order of C_org_ loss was T_1_>T_2_>T_3_>T_4_. Thus the maximum C_org_ loss of 35.31% occurred in T_1_ and this loss is higher than the losses recorded in previous studies; 4.8–12.7% in Suthar (2009), 21–29% in Suthar (2007a) and 12.8–27.2% in Suthar and Singh (2008a).

**Table 4:** Chemical composition of substrates at the end of experimentation (mean ± S.D.; n = 3).

Treatment	pH	C_org_ (g kg^−1^)	N_tot_ (g kg^−1^)	P_avail_ (g kg^−1^)	C:N ratio	Ca_exch_ (g kg^−1^)	Fe (mg kg^−1^)	Pb (mg kg^−1^)	Al (mg kg^−1^)
T_1_	7.13 ± 0.01ab	106.00 ± 1.73bc	28.93 ± 0.40b	30.87 ± 0.63b	3.66 ± 0.02b	49.57 ± 0.55a	208.60 ± 0.55a	15.21 ± 0.16a	58.11 ± 0.24a
T_2_	6.93 ± 0.06c	103.67 ± 3.06c	28.83 ± 1.31b	30.19 ± 0.30b	3.60 ± 0.09b	56.89 ± 0.12b	205.30 ± 0.80a	16.05 ± 0.10b	60.63 ± 0.17b
T_3_	7.07 ± 0.06b	111.33 ± 1.53b	26.60 ± 2.95b	30.66 ± 0.08b	4.21 ± 0.40b	48.50 ± 0.36c	219.40 ± 0.53b	18.11 ± 0.10c	55.07 ± 0.50c
T_4_	7.23 ± 0.02a	209.24 ± 0.90a	25.57 ± 1.30a	27.28 ± 0.73a	8.20 ± 0.44a	47.68 ± 0.52d	238.40 ± 0.85c	18.59 ± 0.24d	66.46 ± 0.52d

Mean values followed by different letters are statistically different (ANOVA, Dunns multiple-ranged test; P < 0.05.

Characteristics of fecal sludge, organic soils and bulking material used for experimentation (mean ± S.D.; n = 3) are presented in Table 1.

Higher C_org_ mineralization signifies better stabilization and results recorded in present study could be due to the effect of the substrate enrichment with the black soil combined with the coconut coir which could have provided a superior substrate media for the earthworms to thrive in the mineralization of the organic carbon. C_org_ mineralization is more effective when the carbon-to nitrogen ratio is controlled and maintained in substrates (Chang and Chen, 2010; Batham et al., 2013). The black soil combined with the coconut coir could have provided this function because it has been reported that organic sourced bulking materials play an important role in controlling the air supply, moisture and other important composting parameters that affect the carbon-to nitrogen ratio (Adhikari et al., 2008).

N_tot_ content in the vermicompost was enhanced (Table 4). The results are in accordance with previous studies (Ghosh et al., 2007; Thomas et al., 2013; Rupani et al., 2013; Atiyeh et al., 2000) which demonstrated that vermicomposting of organic material have a considerable impact on nitrogen enhancement in the vermicompost produced. However, the order of N_tot_ enhancement in the vermicompost was T_1_ (25.10%)> T_2_ (8.70%)> T_3_ (17.18%)> T_4_ (4.22%), indicating that the vermicompost produced from T_1_ had the maximum N_tot_ enhancement. This could be due to the additional N supplied by both the black soil and the coconut coir and released into the vermicompost. Nevertheless, a Spearman correlation matrix revealed a strong negative correlation (coefficient = -0.820, n = 3, p < 0.001) between the C_org_ loss and N_tot_ enhancement. This correlation result could be due to the fact that as earthworms and composting microbes effect mineralization of the C_org_, they concurrently enrich the organic material with nitrogen through the addition of their excretory products, mucus, body fluid, enzymes and even by decaying worm tissue after death to the substrate (Suthar, 2009).

Chemical compositions and metal contents of substrates at startup used for experimentation (mean ± S.D.; n = 3) are presented in Table 2.

The vermicompost produced from the treatments also had P_avail_ enhanced and this was consistent with results from previous studies (Wira et al., 2011; Kala et al., 2009; Khan et al., 2014). P_avail_ increase was in the order T_1_ (34.14%)> T_2_ (16.67%)> T_3_ (6.46%)> T_4_ (3.73%) and statistically, the vermicompost produced showed a significant difference between treatments P_avail_ (F = 490.4, P < 0.001). P_avail_ enhancement in the vermicompost could be due to the passage of the organic matter through the gut of earthworms resulting in the release of phosphorus in the available forms performed partly by earthworm gut phosphatases which is further enhanced by P-solubilizing microorganisms present in worm casts (Suthar, 2009). T_1_ recording the maximum P_avail_ enhancement could be due to the additional P released by both the black soil and coconut coir into the vermicompost. Results on nutrient enhancement (N_tot_ and P_avai_) in the present study therefore support the hypothesis that substrate enrichment with soil combined with plant derived organic materials provides a superior substrate to produce quality vermicompost rich in nutrients for agricultural reuse.

The concentration of Ca_exch_ was also higher in the vermicompost produced (Table 4) compared to the initial values (Table 2). Ca_exch_ increase showed significant differences (F = 1220, P < 0.001) between treatments. Ca_exch_ increase could be attributed to earthworm activities that changes proportions of Ca from bound forms in substrates to free and exchangeable forms which can be easily absorbed unto the vermicompost. Thus, when organic material passes through the gut of the earthworm, calcium oxalate crystals get converted to calcium bicarbonate, which consequently enriches the vermicompost with higher Ca concentration (Rupani et al., 2013). Vermicompost rich in Ca_exch_ is one of the finest source of fertilizer for agricultural crops and agricultural lands deficient in Ca (Bettiol, 2004).

The C/N of the vermicompost decreased compared to the initial values in the order: T_1_>T_3_>T_2_>T_4_ (Table 4). Results of C/N decrease in the vermicompost is similar to previous studies (Ghosh et al., 2007; Kala et al., 2009; Huang et al., 2006). It has been explained that, the simultaneous loss of C_org_ and the enhancement of N_tot_ contributes to the reduction of C/N in vermicompost (Suthar and Singh, 2008b). Reduced C/N reflects a satisfactory degree of organic waste stabilization and maturity (Senesi, 1989). The control with no enrichment produced vermicompost with the least C/N (Table 4) and this demonstrated that substrate enrichment with plant derived organic materials combined with agricultural soil is effective to increase the maturity and stabilization of vermicompost. C/N is one of the most widely used indices to assess the level of maturity and stabilization of vermicompost (Huang and Wong, 2004).

The vermicompost produced at the end of study contained lower concentrations of Fe, Pb and Al (Table 4) than the initial values (Table 4) and the decreases (% of their initial values) were in the range: 17.31–21.95% for Fe, 3.89–19.60% for Pb and 14.95–49.45% for Al.

The reduction of metals in the vermicompost is similar to that observed in previous studies (Maboeta and Rensburg, 2003; Suthar, 2009). Heavy metal removal in vermicompost of the present study however did not exhibit a direct relationship between earthworm's activities and metal loss from substrates. Metals removal in vermicompost may be due to the digestion and assimilation of the organic waste by the earthworms as they are capable of ingesting and accumulating some amount of heavy metals in their body biomass (Suthar et al., 2007). Further analysis of the earthworm tissues is however needed to confirm this assertion.

Microbial biomass and enzyme contents of substrates used for experimentation (mean ± S.D.; n = 3) at startup is presented in Table 3.

Chemical compositions and metal content of substrates after 12 weeks of experimentation (mean ± S.D.; n = 3) are shown in Table 4.

### 3.2 Biological and biochemical changes during vermicomposting

**Figure 1:**
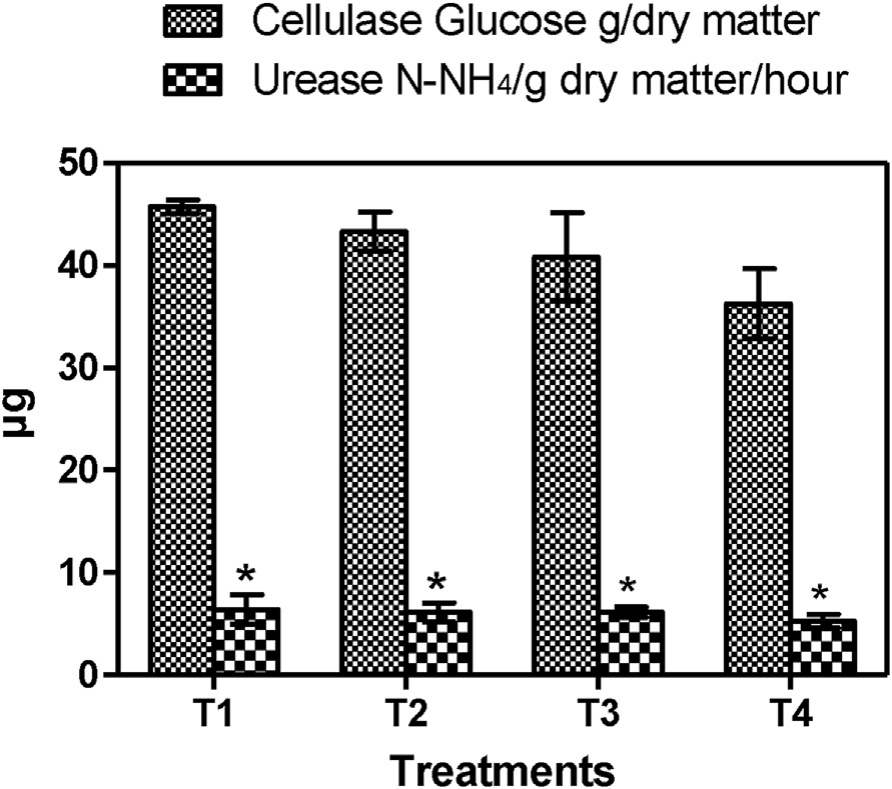
Enzyme concentration of substrates at the end of experimentation. The symbol star (∗) indicates the significant difference between control and experiment vermibed.

Microbial biomass abundance varied in the vermicompost produced from the treatments (Figure 1). There was a significant enhancement in the microbial carbon (μg C/g dry mass) (F = 355.6, p < 0.0001) and the microbial nitrogen (μg N/g dry mass) (F = 125.2, p < 0.0001). The microbial biomass enhancement in the vermicompost was however in the order T_1_ (160.40%)> T_2_ (123.20%)> T_3_ (120.30%)> T_4_ (74.20%) for biomass carbon and T_1_ (146.40%)> T_2_ (114.40%)> T_3_ (110.20%)> T_4_ (70.60%) for biomass N. Results in present study corresponds with previous studies by Aira et al. (2002) and Fernandes et al. (2005) which indicate that the microbial biomass could be due to the earthworm's burrowing and casting activities in the substrate, secreting mucus and other substances to change the physico-chemical composition. These activities provide suitable conditions for the microcosm under which the composting microbes are stimulated to multiply. This result generally in a microbial biomass boost and accelerate the rates of organic matter decomposition, humification and nutrient release (Lavelle et al., 1993). Barois and Lavell (1986) also explained that total microbial biomass is generally enhanced in their guts of earthworms in a mutualistic digestion process where ingested composting microbes are multiplied and stimulated to a higher level of activity by mucus secretion in the foregut of the worms.

The control treatment produced vermicompost with the least microbial biomass enhancement (Figure 1) and this could be attributed to the absence of soil in the substrate. Results indicated that microbial biomass was most enhanced in vermicompost produced from T_1_ treatment. The black soil for the substrate enrichment in T_1_ did not only modify the physical properties of the substrate, but may also have changed the biodegradation kinetics and the chemical composition by releasing most of the additional nutrients required to boost microbial abundance (Adhikari et al., 2008). Fernandes et al. (2005) further explains that microbial biomass in a vermicomposted material reflect the initial concentration of nutrients available and released into substrates to stimulate the activities of the microcosm. Therefore, the behavior of the microcosm and the microbial biomass in a vermicomposting system largely depend on the quality of the organic material used to enrich substrates.

A Pearson correlation matrix of earthworm mortality to the microbial biomass abundance demonstrated strong positive correlations (Pearson r = 0.9813, p = 0.0187 and Pearson r = 0.9844, p = 0.0156 for microbial biomass-C and microbial biomass-N respectively). These correlations disclosed the fact that the microbial biomass heightened with earthworm mortality rise and this corroborates similar reports from previous studies (Edwards and Bohlen, 1996; Aira et al., 2002; Zhang et al., 2000; Tiunov and Scheu, 2000; Flegel and Schrader, 2000) that indicate that earthworm presence in substrates affects microbial abundance through direct feeding of the microorganisms. Results of present study are also in line with previous studies (Tognetti et al., 2013; Atiyeh et al. 2000; Zhang et al., 2000) that explains that the presence of earthworm species in substrates results direct consumption of microorganisms by the earthworms. This ends up affecting the microcosm directly and indirectly (Brown, 1995). Tiunov and Scheu (2000) added that the rate of direct consumption of microorganisms by earthworms is highly dependent on the kind of nourishment needed and supplied by the substrate. Several authors (Levanon and Pluda, 2002; Tiquia et al., 2002) have therefore suggested that in order to accelerate the augmentation of the microcosm and the microbial biomass essential for agricultural use, produced vermicompost needs to be passed through a thermophilic phase to renew the microbial communities.

There was enzyme (cellulase and urease) concentration enhancement in the vermicompost produced at the end of the study (Figure 1) compared to the initial (Table 3). These results relate well with previous studies (Fernandes et al., 2005; Melo and Marques, 2000; Albiach et al., 2000). Enzyme enhancement could be due to the passage of the ingested material through the guts of earthworms which stimulate the secretion of the digestive enzymes, ie. cellulase and urease, along with intestinal mucus which mixes with the egested vermicompost (Vahed et al., 2011). The order of enzyme enhancement was T_1_ (172.20%)> T_2_ (162.20%)> T_3_ (160.50%)> T_4_ (113.47%) for Cellulase and T_1_ (76.30%)> T_2_ (68.32%)> T_3_ (64.20%)> T_4_ (46.50%) for Urease. Likewise, the least enzyme concentration enhancement was found in vermicompost produced from the control treatment (T_4_) and the most enhancement produced from T_1_. This demonstrated that the use of soil to enrich substrates largely influenced the enzyme concentration in the vermicompost. Also the black soil treatment producing vermicompost with the most enzyme concentration could be due to the release of most additional nutrient fraction needed for the vermicomposting process that is solely responsible for the enzyme enhancement (Yurekli, 2010). Notwithstanding, there was a positive significant correlation between the microbial biomass rise and the enzyme enrichment (coefficient = 0.64; p < 0.05) and this establishes the fact that the enzyme concentrations provided indications for the changes in the microbial biomass of the vermicomposted material.

Figure 1 presents enzyme concentration in the vermicompost at the end of the study (mean ± S.D, n = 3). The results show that enzyme concentration increased in the end product after 12 weeks vermicomposting.

**Figure 2:**
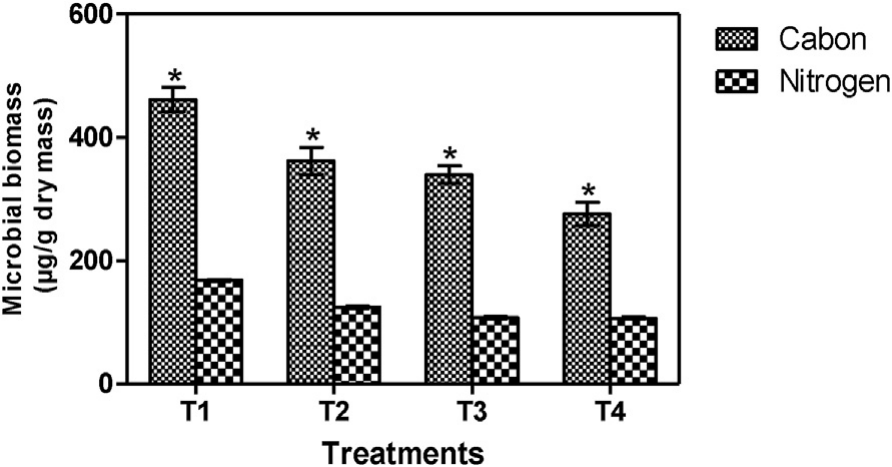
Microbial biomass of substrates used for experimentation (mean ± S.D.; n = 3) at the end of experimentation. The symbol star (∗) indicates the significant difference between control and experiment vermibed

Figure 2 presents microbial biomass in the vermicompost at the end of the study (mean ± S.D, n = 3). The results show that microbial biomass increased in the end product after 12 weeks vermicomposting.

### 3.3 Earthworm growth and reproduction performance in different treatments

Results showed that earthworm mortalities recorded less than 16% in all treatments except the control (T_4_) which recorded 38.33 ± 13.74% (Table 5). Results of earthworm mortality in treatments is lower than that recorded in previous studies (Atiyeh et al., 2000; Domínguez et al., 2000) and this could be due to the aerobic condition of the enriched substrate since anaerobic conditions are a major source of worm mortality (Edwards, 1995). This demonstrated that the substrate technique used in the present study did not only accelerate the waste stabilization but at the same time maintained the mortality rate below 16%.

**Table 5:** Growth and reproduction performance of *E. fetida* in different treatments (mean ± S.D., n = 3).

Treatment	Total earthworm mortality at the end of experiment (%)	Initial individual live weight (mg)	Individual weight gain (%)	Total individual live weight (mg)	Biomass gain (mg)	Individual growth rate (mg day^−1^)	Reproduction rate (cocoonsworm^−1^ day^−1^)
T_1_	8.33 ± 5.77bd	281.42 ± 0.71a	107.33 ± 3.21bd	583.46 ± 7.56bd	302.04 ± 2.20bd	5.39 ± 0.15bd	0.067 ± 0.011b
T_2_	11.67 ± 2.89bc	281.75 ± 0.47a	96.00 ± 5.00bc	552.24 ± 14.14c	270.48 ± 4.10bc	4.83 ± 0.25bc	0.055 ± 0.008b
T_3_	15.00 ± 5.00b	281.55 ± 0.57a	97.00 ± 4.36b	554.64 ± 11.77b	273.09 ± 2.01b	4.88 ± 0.21b	0.054 ± 0.005b
T_4_	38.33 ± 13.74a	281.89 ± 0.35a	66.33 ± 2.08a	468.88 ± 5.38a	186.99 ± 5.10a	3.34 ± 0.10a	0.012 ± 0.01a

Mean values followed by different letters are statistically different (ANOVA, Dunns multiple-ranged test; P < 0.05.

**Figure 3:**
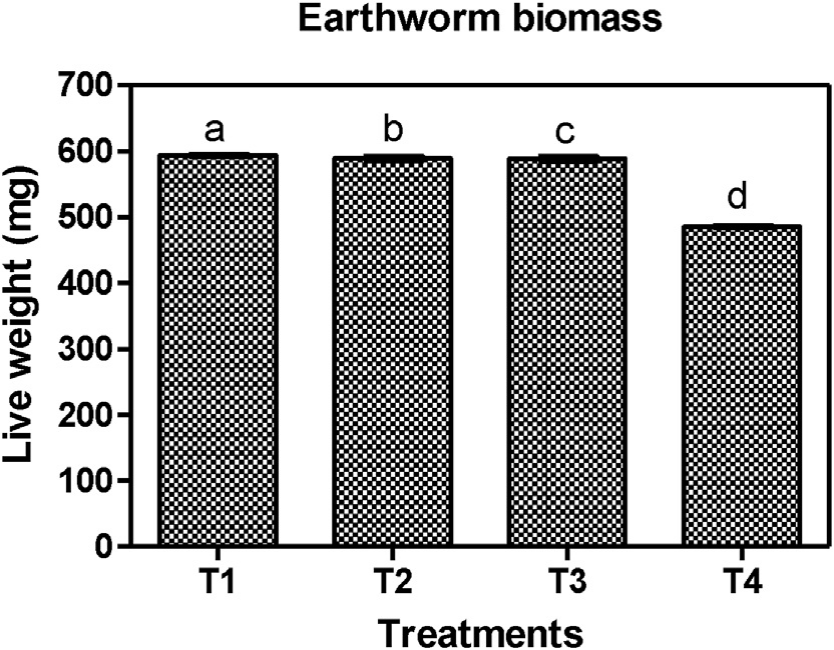
Earthworm biomass at the end of the experimentation. The significant difference (p < 0.05) is indicated by different letters.

**Figure 4:**
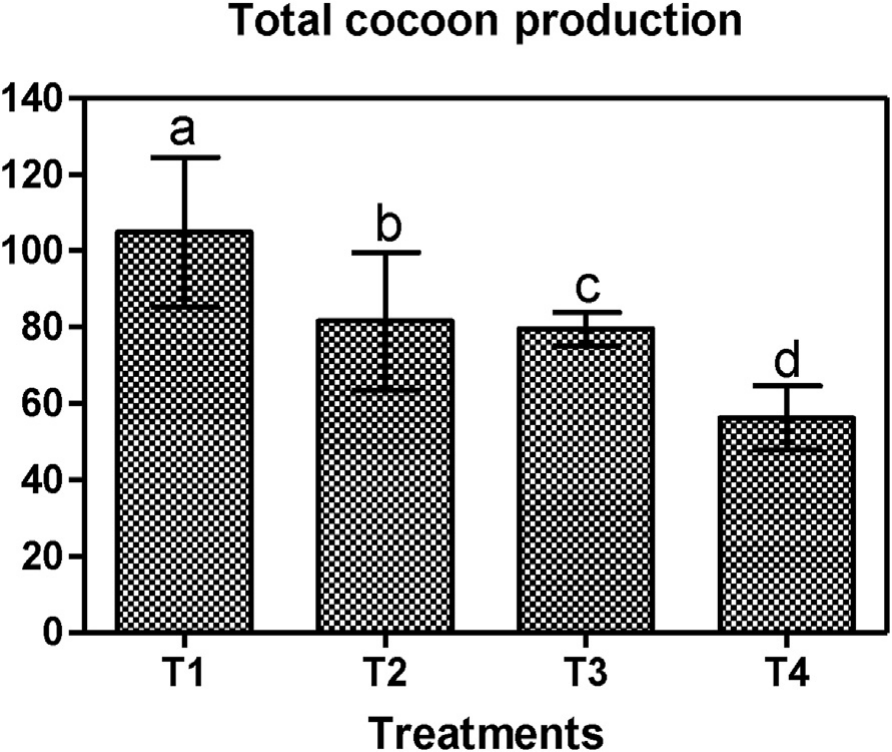
Total cocoon production at the end of experimentation. The significant difference (p < 0.05) is indicated by different letters.

**Figure 5:**
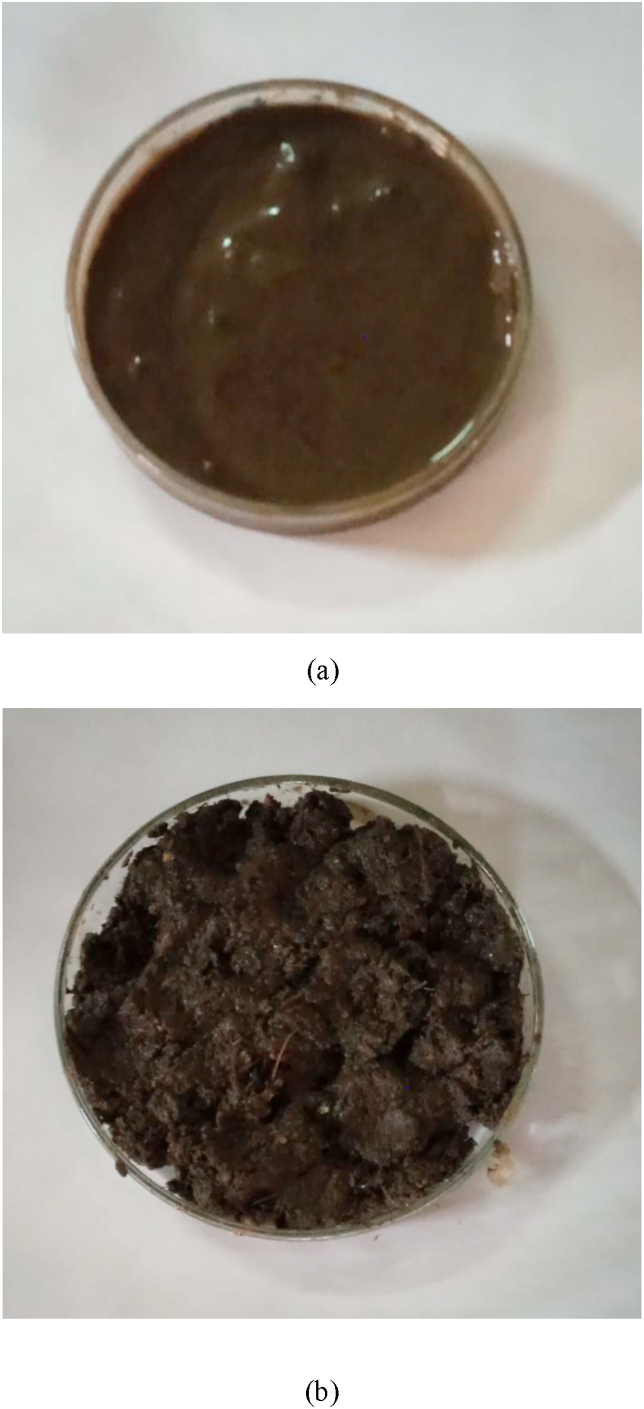
(a) Raw fecal sludge and (b) Vermicompost sample.

The growth and reproduction of *E. fetida* increased at the end of the study and this is in accordance with previous studies (Ndegwa et al., 2000; Garg and Kaushik, 2005; Maboeta and Rensburg, 2003). The vermiculture parameters of *E. fetida* in the present study demonstrated significant differences between treatments in terms of individual weight gain (%) (F = 63.71, p < 0.0001), total individual live weight (mg) (F = 68.84, p < 0.0001), biomass gain (mg) (F = 66.39, p < 0.0001), individual growth rate (mg day^−1^) (F = 66.71, p < 0.0001) and reproduction rate (cocoonsworm^−1^ day^−1^) (F = 29.31, p = 0.0001). The individual live weight of *E. fetida* increment at the end of the study was in the order T_1_ > T_2_ > T_3_ > T_4_. Similar trend was observed with the individual growth rate where the maximum and minimum individual growth rate (mg day^−1^) occurred in T_1_ and T_4_ respectively (Table 5). *E. fetida* also showed the maximum cocoon production in T_1_ followed by T_2_ and T_3_ with the least occurring T_4_. This demonstrated that the substrate enrichment technique used in the present study positively had a significant effect on the earthworms' vermiculture and t could be attributed to the release of additional nutrients from the materials used in the substrate enrichment. Moreover, Suthar (2009) reported that organic material plays an important role in earthworm's growth due to the direct consumption, digestion and assimilation to add to their body biomass and the indirect use of the organic material for microcosm boost which provides extra nourishment to the worms. However, the maximum vermiculture development recorded in T_1_ (Figure 3 and Figure 4) could signify that the black soil could have provided the most nourished enriched substrate needed for the vermiculture. The appropriate policies this study recommends based on findings is that compost producers that seek to produce vermicompost from fecal sludge should include organic soils in preparing the substrates because the use of organic soils in substrate enrichment has been found in this study to greatly improve the quality of the vermicompost and also contribute greatly to the reduction in the earthworm mortality (see Figure 5).

Growth and reproduction performance of *E. fetida* in different treatments (mean ± S.D., n = 3) is presented in Table 5.

Figure 3 present the biomass production rate in *E. fetida* collected from different treatments. Results show that earthworm biomass increased in enriched substrates at the end of 12 weeks of vermicomposting.

Figure 4 present the cocoon production rate (b) in *E. fetida* collected from different treatments. Results show that cocoon production increased in enriched substrates at the end of 12 weeks of vermicomposting.

## 4 Conclusions

The study revealed that substrate enrichment technique of using organic soils combined with coconut coir is effective in producing highly nourished and stabilized vermicompost and this technique is also suitable for the vermiculture of *E. fetida*. Thus, the vermicompost produced from using black soil, red laterite soil and sandy soil combined with coconut coir were rich in nutrients, had lower levels of metals, had higher levels of microbial biomass and enzyme concentration. However, black soil treatment produced the most stabilized and nourished vermicompost which was attributed to the physico-chemical properties and the release of additional nutrients by the substrate treatment. The study has demonstrated that the substrate enrichment treatments provided a suitable microclimate condition for microcosm development for vermicompost production and *E. fetida* vermiculture. The nutritious nature and the reduced metal concentration of the produced vermicompost might be safe and productive for agricultural use. However, the effectiveness of the vermicompost on plant growth would need further investigation. Also, a more comprehensive study on the evaluation of metal accumulation in the body tissues of earthworms as a bioindicator of a potential metal contamination is needed to establish a strong hypothesis of using this substrate enrichment technique for vermiculture.

## Declarations

### Author contribution statement

Rapheal Nsiah-Gyambibi: Conceived and designed the experiments; Performed the experiments; Analyzed and interpreted the data; Contributed reagents, materials, analysis tools or data; Wrote the paper.

Helen Michelle Korkor Essandoh; Nana Yaw Asiedu; Bernard Fei-Baffoe: Analyzed and interpreted the data; Contributed reagents, materials, analysis tools or data; Wrote the paper.

### Funding statement

This work was supported by the Regional Water and Environmental Sanitation Centre Kumasi(RWESCK) at the 10.13039/501100012015Kwame Nkrumah University of Science and Technology (KNUST), Kumasi with funding from the Ghana Government through the World Bank under the Africa Centres of Excellence project. The views expressed in this paper do not reflect those of the World Bank, Ghana Government and KNUST.

### Data availability statement

Data included in article/supplementary material/referenced in article.

### Declaration of interests statement

The authors declare no conflict of interest.

### Additional information

No additional information is available for this paper.
